# A17, the First Sequenced Strain of Lactococcus lactis subsp. cremoris with Potential Immunomodulatory Functions

**DOI:** 10.1128/genomeA.01563-14

**Published:** 2015-02-12

**Authors:** Chih-Hsien Yang, Chien-Chen Wu, Wei-Shen Cheng, Ming-Chuan Chung, Ying-Chieh Tsai, Chuan-Hsiung Chang

**Affiliations:** a Institute of Biomedical Informatics, National Yang-Ming University, Taipei, Taiwan; b Center for Systems and Synthetic Biology, National Yang-Ming University, Taipei, Taiwan; c Institute of Biochemistry and Molecular Biology, National Yang-Ming University, Taipei, Taiwan; d Probiotics Research Center, National Yang-Ming University, Taipei, Taiwan

## Abstract

Lactococcus lactis subsp. cremoris A17, isolated from Taiwan fermented cabbage, is the first sequenced strain of L. lactis subsp. cremoris with immunomodulatory activity and antiallergic functions. The resulting A17 draft genome contains 2,679,936 bp and indicates that A17 is a potential exopolysaccharide-producing strain without any known virulence gene.

## GENOME ANNOUNCEMENT

Lactococcus lactis subsp. cremoris strains are widely used in fermented food production, but no sequenced L. lactis subsp. cremoris strain has been reported to have immunomodulatory effects. Several L. lactis strains were reported to induce cytokine production in the J.774.1 mouse cell line and ovomucoid-specific responses in mice (1). In vaccination applications, the strain L. lactis D1813 isolated from kuruma shrimps induced IFN-gamma production and helped to resist the infection of Vibrio penaeicida (2). Most L. lactis subsp. cremoris strains were sequenced for studying their fermentation functions in food production, but none for their immunomodulatory activities and host protection functions.

L. lactis subsp. cremoris A17 (DSM 27109, abbreviated hereafter as A17) isolated from Taiwan fermented cabbage, was reported to induce IFN-gamma production in human peripheral blood mononuclear cells, suggesting an immunomodulatory activity toward the T-helper cell type 1 response (3). Furthermore, oral administration of live or heat-killed A17 to ovalbumin-sensitized BALB/c mice reduced the production of serum IgE and splenocytic cytokines associated with allergic response (3). In this report, we sequenced the genome of A17 as the first reference to study the underlying mechanism and genetic elements of immunomodulatory activity and host protection functions of L. lactis subsp. cremoris strains.

The genome of A17 was sequenced with both MiSeq (Illumina, Inc.) and RSII (Pacific Biosciences of California, Inc.) platforms to generate 4,117,596 paired-end reads and 163,471 PacBio long reads, respectively. We trimmed Illumina paired-end reads by limiting the quality score of reads to more than 30 and removed 20 nucleotides on both ends of the reads. The filtered Illumina reads were *de novo* assembled (with a word size of 50 bp and a bubble size of 98 bp) with PacBio long reads as the guidance in CLC Genomics Workbench 7.04 (Qiagen) with the CLC Microbial Genome Finishing Module (Qiagen). The resulted genome assembly of A17 has 16 contigs, comprising 2,679,936 bp. The estimated G+C content of the assembled A17 genome is 35.6%. The A17 genome assembly was annotated using the NCBI prokaryotic genome annotation pipeline (PGAP) and then uploaded to NCBI GenBank. The annotation of the A17 assembly contains 2,500 genes, including 2,321 coding DNA sequences (CDS), 93 pseudogenes, 18 rRNA genes (5S, 16S, and 23S), 67 tRNA genes, one ribozyme gene (RNase P), and 63 frameshifted genes. A17 was shown to be a potential exopolysaccharide (EPS) producing bacterium with seven genes for polysaccharide metabolism found in the genome. These genes encode one polysaccharide deacetylase (JL36_00195), one polysaccharide permease (JL36_11180), one membrane protein for polysaccharide transport (JL36_07670), and 4 proteins for polysaccharide biosynthesis (JL36_05435, JL36_09505, JL36_09510, and JL36_11200). After a BLASTn search (with default parameters) against the virulence factors database (VFDB [see http://www.mgc.ac.cn/VFs/]), none of the genes in the A17 genome were found to be similar to virulence genes in VFDB.

### Nucleotide sequence accession numbers.

This draft genome of L. lactis subsp. cremoris A17 has been deposited in GenBank under the accession no. JQIC00000000. The version described in this paper is the first version, JQIC01000000.
